# A New Recognition Method for the Auditory Evoked Magnetic Fields

**DOI:** 10.1155/2021/6645270

**Published:** 2021-02-09

**Authors:** Yulong Feng, Wei Xiao, Teng Wu, Jianwei Zhang, Jing Xiang, Hong Guo

**Affiliations:** ^1^State Key Laboratory of Advanced Optical Communication Systems and Networks, Department of Electronics and Centre for Quantum Information Technology, Peking University, Beijing 100871, China; ^2^School of Physics, Peking University, Beijing 100871, China; ^3^MEG Centre, Division of Neurology, Cincinnati Children's Hospital Medical Centre, Cincinnati, Ohio, USA

## Abstract

Magnetoencephalography (MEG) is a persuasive tool to study the human brain in physiology and psychology. It can be employed to obtain the inference of change between the external environment and the internal psychology, which requires us to recognize different single trial event-related magnetic fields (ERFs) originated from different functional areas of the brain. Current recognition methods for the single trial data are mainly used for event-related potentials (ERPs) in the electroencephalography (EEG). Although the MEG shares the same signal sources with the EEG, much less interference from the other brain tissues may give the MEG an edge in recognition of the ERFs. In this work, we propose a new recognition method for the single trial auditory evoked magnetic fields (AEFs) through enhancing the signal. We find that the signal strength of the single trial AEFs is concentrated in the primary auditory cortex of the temporal lobe, which can be clearly displayed in the 2D images. These 2D images are then recognized by an artificial neural network (ANN) with 100% accuracy, which realizes the automatic recognition for the single trial AEFs. The method not only may be combined with the source estimation algorithm to improve its accuracy but also paves the way for the implementation of the brain-computer interface (BCI) with the MEG.

## 1. Introduction

Magnetoencephalography (MEG) utilizes extremely sensitive magnetic sensors, such as optical pumped atomic magnetometers (OPMs) and superconducting quantum interference devices (SQUIDs), to capture the feeble signal originated from the brain and enable researchers to investigate neuronal activities [1–4]. MEG is a frontier tool in scientific research and clinical application. On the one hand, it can be employed to divide and study different brain functional areas [5, 6]. On the other hand, MEG is also an effective method for clinical diagnoses and treatments of some brain functional diseases, such as mild traumatic brain injury [7, 8] and autism spectrum disorder [9], and provides novel insights into the biological mechanisms underlying some brain disorders such as dementia [10], depression [11], and psychosis [12]. Especially for epilepsy, MEG allows us to locate the epileptic foci without risky invasion procedures [13].

The signal generated by the brain in response to different stimuli is the hot topic in the study of the brain function. These signals are called event-related potentials (ERPs) [14] in the electroencephalography (EEG) or event-related magnetic fields (ERFs) in the MEG, including sensory ERFs, motor ERFs, long latency, and artifacts [15]. In the EEG, the amplitudes, phases, waveforms, occurrence times, and source locations of ERPs are the major characteristics to be studied [16, 17]. It is also important to explore the dependences of the ERPs with different subjects [18, 19] or with different stimulus [20–23]. Thus, MEG is an edge tool in clinical setting, such as brain functional diseases, biomedical engineering, and medical devices [24–27]. In the context of given stimuli, studying features of the ERPs is helpful for obtaining the information from different brain functional areas [28, 29]. On the contrary, with knowledge of the brain functional regions, the recognition for different ERPs can help us understand the stimuli the subjects are exposed to [30, 31] and the mental states the subjects are in [32]. It is the basis of converting the different brain responses of the subjects into different behaviours, which is also the goal the brain-computer interface (BCI) intends to achieve [33–35]. Meanwhile, ERFs takes the form of magnetic induction intensity of brain responses reflected by ERPs. The recognition for the ERFs would also provide complementary information for MEG application that is important for brain research.

Due to the weakness and fuzziness of the ERFs and existence of various noises [36], even with the magnetic shielding room (MSR), the single trial ERFs cannot be precisely observed. We need to stimulate the subjects hundreds of times and get enough single trial ERFs to average them precisely. Averaging could suppress the random noise and strengthen the pattern of the ERFs, which is a conventional data processing method for the ERPs in the EEG. However, the average signal depends on the common patterns and components existing in the single trial ERFs, which may vary widely in both the time domain and the scalp distribution. These variations result from different strategies employed by the subject for processing stimuli. The physiological differences always appear in the subject's performance during each stimulus, such as expectation, attention, arousal, alertness, and fatigue [31, 37, 38]. At the same time, signals generated by ongoing activities of the subject, which are unrelated to the event of interest, compete with the ERFs for the signal space and play the role of noise [31]. These signals appear randomly and irregularly and are seldom well defined, which makes the positive identification of single trial signal very difficult, while it does not mean that these signals are useless. For example, these signals are usually required to calculate the noise matrix in the process of magnetic source imaging (MSI) [39–41]. Besides, if a considerable noise is introduced in a single trial, averaging would also bring it into the average signal. Averaging disregards some information of the interested signal contained in the single trial and can only produce a signal prototype that is not representative of any of the single trials induced in the average, which makes the recognition and determination of the single trial ERFs an important problem in brain science [31, 42].

Compared to averaging, direct recognition for the single trial ERFs does not need to store the data, providing the possibility to perform the on-the-fly identification. In the help of this advantage, a door opens for the study of cognitive brain function, which is a hot spot of experimental psychophysiology [43]. Cognitive variables like visual, auditory, sensory, and even emotional changes, as well as psychological changes, which vary from trial to trial, may be manipulated and sorted out in the study, showing the possibility that the ERFs could be used to implement an objective measure of the brain processes implicated in learning and problem solving. Furthermore, if the single trial ERFs can be read from the MEG and translated by the computer into a perceivable behaviour, the specific brain activity resulting from the specific consciousness can generate the specific action, which is exactly what BCI wants to achieve [44, 45].

Current single trial recognition methods are mainly for the ERPs in the EEG. Independent component analyses (ICA) are first employed to separate the feature vectors from the single trial ERPs to represent their characteristics [42, 46]. The original detected EEG signal is the result of multiple factors, such as the diversity of ERPs' sources, the inconsistency in the electrical conductivity of brain tissues, and differences between sensors, leading to the separated characteristics being not evident. With the improvement of instruments and detection methods, the signal waveforms in the EEG become a starting point to solve the single trial ERPs recognition problem. Various statistics are constructed using amplitudes [15], phases [31], and frequencies [30, 47, 48] to detect different ERPs. However, signals detected by the MEG and the EEG are different [49]. Data processing methods that are suitable for the EEG signal may not be appropriate for the MEG signal. The MEG signal has less interferences and more sensors can be used to get the location information. Besides, there are some alternative techniques such as soft computing capable of studying magnetic fields [50–52]. With the application of machine learning algorithms in medicine [53–55], in this work, we intend to enhance the position features indicating the spatial distribution of the MEG signal and utilize the artificial neural network (ANN) to recognize the single trial auditory evoked magnetic fields (AEFs), which are called auditory evoked potentials (AEPs) in the EEG. Two AEFs datasets and one noise dataset are used to verify recognition method of the AEFs. After enhancing the position features with the signal enhancement method, these position features are also highlighted as 2D images and are automatically recognized with GoogLeNet [56].

In this article, we first describe the AEFs dataset, including the data collection and the data format, in Materials and Methods. Then the signal enhancement and recognition method for the single trial AEFs are described in detail. Results show the effect of the signal enhancement method and the recognition results for the single trial AEFs. In Discussion, we further discuss the advantages and disadvantages of this single trial AEFs recognition method compared with traditional methods and prospect the future development and application of the method. At last, we summarize this article in Conclusions.

## 2. Materials and Methods

### 2.1. Data Description

**Figure 1:**
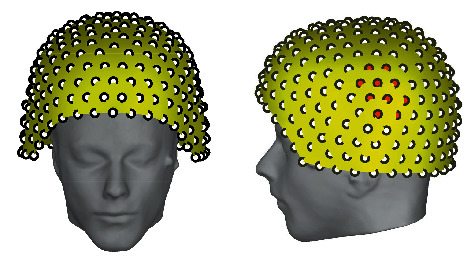
A map of all channels of the SQUID-MEG system drawn with the MEG data processing software Brainstorm. This system is employed in the experiment to measure the MEG signal. The red dots indicate the channels distributed in the auditory area of the temporal lobe.

The data used in this work comes from the open-source database on the Brainstorm website [57]. The experiment with the method involves three datasets. The first and second datasets are AEFs data and the third dataset is the noise recorded in the same empty room. These data are all recorded with a SQUID-MEG device, which is produced by CTF corporation, Canada, and the distribution of its sensors is shown in Figure 1. The sensors involved in the data recording are shown in Table 1.

**Figure 2:**
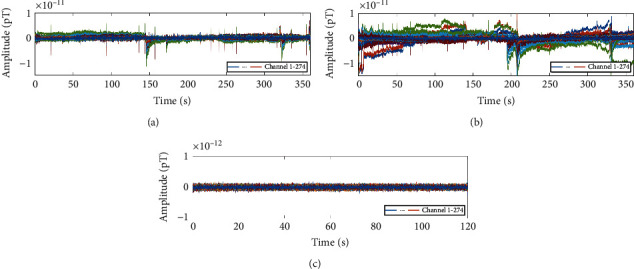
Three datasets used for the single trial AEFs identification. (a) The first AEFs dataset. (b) The second AEFs dataset. (c) The noise dataset. It is the noise recorded in an empty room with the same environment.

Two AEFs datasets are acquired with a sampling rate of 600 Hz in 360 s. The first (second) dataset contains 200 (199) auditory stimuli, which means that it should include 200 (199) single trial AEFs. We use the first dataset as the training data source and the second dataset as the testing data source. The noise collected in an empty room with the same environment is the third dataset. The noise has a length of 120 s and is sampled with a rate of 600 Hz. These three datasets are shown in Figure 2. It should be noted that the spikes that appear in the signal are interferences caused by eye movement signals. In addition, the fluctuations may be caused by various signals from various parts of the brain as well as some environmental noises.

### 2.2. Signal Enhancement

One cannot observe any trace about the single trial AEFs from Figure 2, due to the fuzziness of the single trial AEFs and the existence of various noises. It is necessary to enhance the AEFs and suppress the noise as much as possible. We propose a signal enhancement method, which can be used to enhance the position features of the single trial AEFs. The procedures for signal processing are described below.

**Figure 3:**
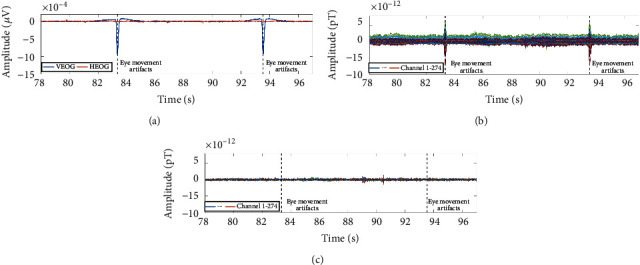
Eye movement artifacts cleaning for the MEG. (a) A segment of eye movement signals measured by the EOG bipolar. The blue line represents the vertical eye movement signal (VEOG) and the red line represents the horizontal eye movement signal (HEOG). The dotted black line shows where eye movement artifacts appear in the time domain. (b) The marked 274-channel MEG signals. (c) The MEG signals after noise cleaning.

Event markers: in order to obtain the single trial AEFs segment, the initial time of the single trial AEFs should be calibrated and marked by the signal recorded from the audio recording channel, because there is a delay between the sound being produced and the sound being heard by the subject, which is about 0.13 s in the experiment, while the appearance time of the stimulus recorded by the audio recording channel is almost the same as the time at which the subjects heard the sound. In addition, the eye movement artifacts and the cardiac artifacts should be marked according to the signals recorded by the bipolar ECG and the bipolar EOG, as is shown in Figure 3(b), so that we can eliminate them with the signal space projection (SSP) algorithm. After the process, the occurrence times of the artifacts and the AEFs are marked in the original detected data.

Preprocessing: the presence of various noises makes the identification of the AEFs difficult. Noise needs to be eliminated as much as possible. Firstly, the SSP algorithm is adopted to remove the eye movement artifacts and the cardiac artifacts [58–61]. Secondly, a 2nd-order infinite impulse response (IIR) notch filter with 3 dB bandwidth of 2 Hz is used to clear *α* and *β* waves of 10 Hz, 11 Hz, 20 Hz, and 21 Hz. At last, an even-order linear phase finite impulse response (FIR) low-pass 40 Hz filter with 60 dB stopband attenuation is performed on the signal to remove as much noise as possible and leave the AEFs. Meanwhile, the line frequency noise and its harmonics (60 Hz, 120 Hz, and 180 Hz) are also filtered. The preprocessing signal is shown in Figure 3(c). After the process, the artifacts, irrelevant MEG signals, and the line frequency noise have been removed.

Interception for the single trial AEFs: in order to obtain the major features of the AEFs, which are three peaks called P50, N100, and P200, respectively (see Appendix A for detailed information), we need to intercept the single trial AEFs. The single trial AEFs last for about 0.3 s and can be intercepted according to the stimulus previously marked in the MEG signal. During the first (second) set of data recording, we stimulate the subject with 200 (199) audio stimuli. Therefore, we can obtain 200 (199) single trials AEFs from the first (second) dataset.

Calculation of correlation coefficients of the MEG signal: among 200 single trials AEFs from the first dataset, it could be found that signals detected in the primary auditory area of the temporal lobe are correlated. Thus, the correlation coefficients are used to enhance the single trial AEFs. We set 0.022 × 1.7 m as the radius and define a neighbourhood for each sensor {*S*_*i*_; *i*=1,2,…, 274} (see Appendix B for more details). For simplicity, the sensor in the centre of neighbourhood is called the selected sensor *S*_*i*_^sel^ and its detected signal is the selected signal *X*_*i*_^sel^. Sensors in its neighbourhood are neighbourhood sensors *S*_*j*_^nei^ and their detected signals are neighbourhood signals *X*_*j*_^nei^. The number of the neighbourhood sensors *m* is 5 to 8. The correlation coefficients *c*_*ij*_ between the selected signal and its neighbourhood signals are calculated as [62, 63]

(1) $$\begin{array}{c} c_{ij}=\frac{E\left(X_{i}^{\text{sel}}X_{j}^{\text{nei}}\right)-E\left(X_{i}^{\text{sel}}\right)E\left(X_{j}^{\text{nei}}\right)}{\sqrt{\text{Var}\left(X_{i}^{\text{sel}}\right)\text{Var}\left(X_{j}^{\text{nei}}\right)}}. \end{array}$$

Signal enhancement: it is a conventional way to stack signals of different channels by weighting as a new signal in the EEG [30]. In this work, when the correlation coefficient *c*_*ij*_ between the neighbourhood signal *X*_*j*_^nei^ and the selected signal *X*_*i*_^sel^ is greater than 0.8, we will make the weighted linear superposition of two signals as the new selected signal. Since the correlation coefficient represents the size of components in one variable which are similar to the others, the weight is selected as the correlation coefficient between them. The new selected signal can be obtained as

(2) $$\begin{array}{c} X_{i}^{{\text{sel}}^{\prime }}=\frac{\left[X_{i}^{\text{sel}}+\sum_{c_{j}\geq 0.8}c_{j}X_{j}^{\text{nei}}\right]}{\left(n-1\right)}, \end{array}$$

where *n* represents the number of neighbourhood signals with an absolute value of the correlation coefficient greater than 0.8. Meanwhile, if there were no neighbourhood signals with a correlation coefficient greater than 0.8, we would do nothing with the selected signal; that is, *X*_*i*_^sel′^=*X*_*i*_^sel^. After the enhancement, if the selected signal *X*_*i*_^sel^ consists mostly of the noise, due to its irrelevance to the neighbourhood signals *X*_*j*_^nei^, the new selected signal *X*_*i*_^sel′^ should not be strengthened, while if the AEFs from the brain were detected by the selected sensor, the surrounding sensors could also detect the same component, which means that the correlation between them would be strong and the new selected signal *X*_*i*_^sel′^ would be enhanced.

2D images: since the permeabilities of brain tissues are approximately the same, while the conductivity varies from tissue to tissue, one of the advantages of the MEG is that its detected signal is a better indicator of its source location. Drawing 2D image is a decent way to highlight the spatial distribution of the MEG signal. We calculate the energy of the enhanced signal to draw the 2D image and normalize it for convenience of comparison. A certain kind of ERFs, that is, the single trial AEFs, can be observed and identified. The method should also be applicable for other ERFs including location information, such as visual evoked magnetic fields (VEFs) and somatosensory evoked magnetic fields (SEFs). If the noise also contained spatial correlation, the enhancement method would strengthen the noise. However, as the noise does not have regular spatial distribution, it can further be filtered out by ANN.

### 2.3. Signal Recognition

In the first AEFs dataset, there are a total of 200 single trial AEFs, 3 of which are seriously polluted by the noise, so they are screened out (see Appendix C). The remaining 197 single trial AEFs are training data source. Each intercepted AEFs segment lasts 0.3 s, ensuring it contains P50, N100, and P200 peaks. At the same time, we randomly intercept 200 equal-length segments from the third noise dataset without overlapping. All 397 signal segments are processed with the signal enhancement method and 397 2D images can be obtained as training data. Similarly, 199 single trial AEFs can be obtained from the second AEFs dataset. 200 other equal-length noise segments are also randomly intercepted from the noise dataset. Therefore, a total of 399 2D images can be obtained as testing data.

Pretrained GoogLeNet is utilized to recognize auditory activation patterns in the single trial data. GoogLeNet is a 144-layer convolutional neural network (CNN). The input image is filtered by each layer of the network to get its features. The initial layer is used primarily to identify common features of the image, such as blobs, edges, and colours. The subsequent layers focus on more specific features to divide the images into different categories. For the single trial AEFs recognition problem, 3 layers of GoogLeNet should be readjusted.

The first adjusted layer is the final dropout layer in the network, which aims to prevent overfitting. The original dropout layer randomly sets input elements to zero with a given probability of 0.5, which is set as 0.6 in the new layer. The second one is the last connected layer that decides how to combine the features that the network extracts into class probabilities, a loss value, and predicted labels. In order to retrain GoogLeNet to classify noise and AEFs 2D images, the last connected layer is replaced with a new fully connected layer with the number of filters equal to the number of classes (noise and AEFs). The third adjusted layer is the final classification layer that is utilized to specify the output classes of the network. The classification layer is replaced with a new one without class labels, which will be automatically set as the output classes during the network training. Then, we retrain GoogLeNet for the single trial AEFs recognition problem, which means that it is trained based on the network parameters obtained from pretraining. We set the initial learning rate as 0.0001, which determines the variation range of parameters in the ANN. The epoch is set as 10, which represents how many times ANN is trained with the same set of training data. We use 80% images for training and the remainder for validation. A random seed is set as the default value in Matlab to generate random numbers.

## 3. Results

**Figure 4:**
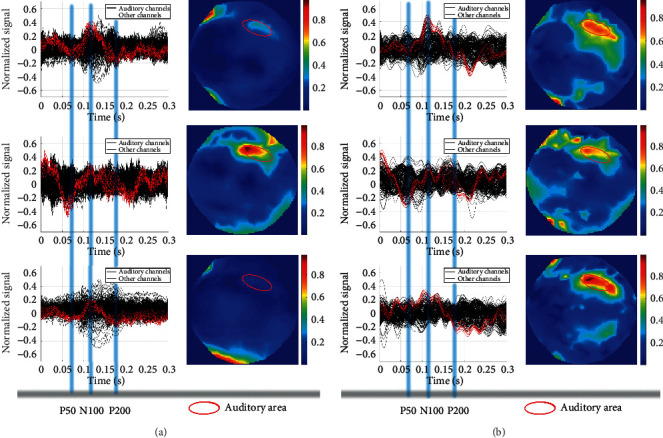
Single trial AEFs enhancement. (a) Three detected original single trial AEFs. The first column displays time domain diagrams of the three AEFs. The *x*-coordinate represents time, and the *y*-coordinate represents the normalized amplitude. The red lines in the time domain diagram represent the signals detected by the auditory channels and the black lines are the signals detected by other channels. The occurrence times of the three peaks P50, N100, and P200 are marked by blue lines. The second column shows the corresponding distribution of the normalized energy across the scalp. The 2D image has a resolution of 224 × 224 pixels so that it can be taken as input by GoogLeNet. (b) Three corresponding enhanced single trial AEFs. The area in the red circle represents the auditory area of the temporal lobe, where the normalized energy is basically concentrated in, which makes GoogLeNet able to easily identify it.

The first three single trial AEFs show the positive effect of the signal enhancement method, as is illustrated in Figure 4. If there were auditory stimuli, the auditory area would send out signals, and the correlation coefficients should be higher than 0.8. The original detected single trial AEFs appear randomly in the time domain and do not have a regular spatial distribution across the scalp. After signal enhancement process, the signals detected by the temporal lobe sensors are all enhanced in the time domain, which makes them become the maximum amplitude or the minimum amplitude of all the channel signals at the P50, N100, and P200 peaks. In the spatial distribution, the normalization energy is concentrated in the auditory area of the temporal lobe of the brain, while the detected signals in other areas tend to be random noises with little correlation, which makes the position characteristics of the AEFs evident. The second detected original AEFs are already well characterized, which indicates that the MEG signal is less interfered during the second measurement. However, the location of the AEFs deviates from the auditory part of the temporal lobe a little. After the enhancement, the deviation has been corrected. It should be noted that, in the experiment, the subjects' left ear is not sensitive to hearing, so in the 2D image, the signal on the right side is more obvious than that on the left side.

**Figure 5:**
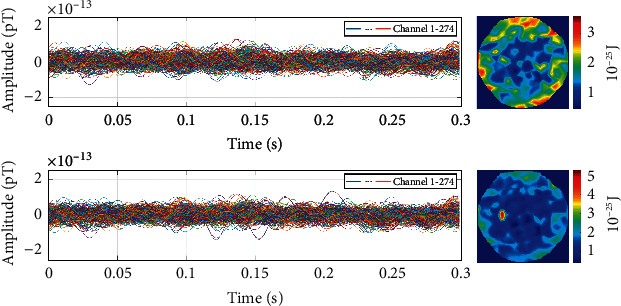
Noise enhancement. The first image shows the original noise in both the time domain and the spatial distribution. The second image shows the processed noise. By comparing two pictures, it can be seen that the signal is concentrated at a point in the front of the brain, which indicates that the source of noise is likely to be located here.

The same operation is implemented for the noise segments to obtain the similar 2D image, as is shown in Figure 5. The noise detected by sensors at various locations appears randomly in space. After the enhancement, most of them are suppressed, but some are also enhanced. For the noise 2D image in Figure 5, it can be speculated that a large magnetic signal fluctuation appears on the front of the brain, resulting in the enhancement of signals in the region, while the signal strength is generally suppressed elsewhere.

After retraining GoogLeNet, it can be utilized to achieve the automatic and on-the-fly recognition for auditory activation patterns in the single trial data. In this work, we use the training data (the testing data), which include 197 (199) single trial AEFs and 200 (200) noise segments, to test the efficiency of GoogLeNet in identifying the single trial AEFs. The recognition accuracies of both datasets are 100%.

## 4. Discussion

In this work, according to the correlation of the signals detected by different sensors, the signal strength is concentrated to the auditory area of the temporal lobe, so that the AEFs can be automatically and timely recognized by ANN. The new method makes full use of the information on the signal spatial distribution contained in the MEG. Although some noises with a specific spatial distribution could also be enhanced, generally the correlation of noises is not strong, and the noise source does not happen to be located in the auditory area. Besides, the method also applies to other ERFs that have a specific spatial distribution, while it may not be suitable for the EEG that is disturbed by different conductivities and measures potential differences.

Compared with the conventional averaging, the new method could preserve the information in the single trial data as much as possible, including the intact ERFs obtained by stimulation and other signals. The single trial recognition can be carried out synchronously with the signal measurement, which means that the real-time identification can be realized. It would provide a powerful tool for psychophysiological study and MEG data processing. At the same time, it is also the basic algorithm to realize the BCI with the MEG. However, the identification of AEFs in single trial requires the AEFs segments so that their position features can be enhanced and displayed, which means that we still need to know the approximate location of the stimulus. If the single trial ERFs can be recognized without any prior condition on the MEG, the information about the external stimulations the body of the subject is exposed to and the mental states the brain of the subject is in could be obtained through the MEG in real time, which is also the true “mind-reading.”

The process of enhancement and recognition for the single trial AEFs is actually the process of extracting and recognizing the position features. It is required that the single trial AEFs are highlighted by the signal enhancement method. In this work, the signal strength is concentrated to its source location, which is the primary auditory cortex of the temporal lobe. On the one hand, if the source of the single trial signal is directly calculated based on some source estimation algorithms such as minimum norm imaging [41], linearly constrained minimum variance (LCMV) beam formers [42], and dipole modelling [43], due to the weakness and fuzziness of the single trial AEFs and the existence of various noises, the results of source estimation may be greatly deteriorated. Nevertheless, if the signal enhancement method proposed in this work is used to process the signal first and then we estimate the source, it may achieve a more accurate result. Further research is needed to figure out how to combine the enhancement method with the source estimation algorithm. On the other hand, ANN is employed here to recognize auditory activation patterns and realize the automatic and on-the-fly recognition for the single trial AEFs. If the training dataset can be extended, ANN should also have the potential to be used to identify VEFs, SEFs, and so forth, which are originated from different functional areas of the brain. It could even be employed to identify the signal amplitude, phase, frequency, and distribution features of some brain diseases such as epilepsy and migraines, realizing the initial diagnosis of these diseases.

## 5. Conclusions

In this work, we propose a new signal recognition method in analysis of the single trial AEFs. ANN can be used to automatically and timely identify the single trial AEFs. This single trial identification can retain the intact original data. Three datasets, two AEFs datasets and one noise dataset, are utilized to experimentally verify the signal enhancement method and the single trial recognition method. Finally, the recognition accuracies of training data and testing data are both 100%. The recognition for the single trial ERFs can not only expand the psychological research methods but also establish the algorithm basis for using the MEG signal to achieve BCI. In addition, it may be combined with the source estimation algorithm to improve its accuracy in the future.

## Figures and Tables

**Figure 6:**
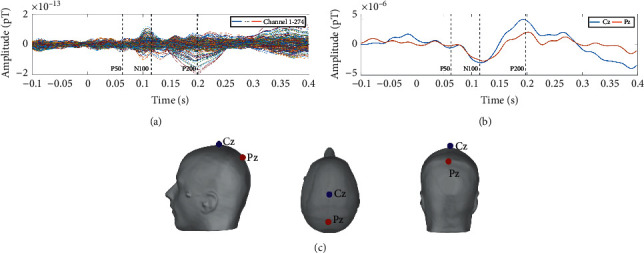
Comparison of the average AEFs in the MEG and the average AEPs in the EEG. (a) The average AEFs in the MEG. By comparison with the AEPs in the EEG, AEFs in the MEG also contain three peaks which are P50 at 68 ms, N100 at 108 ms, and P200 at 193 ms after stimulation, respectively. Time 0 in the picture is the time when the stimulus occurs. (b) The average AEPs in the EEG. There are three distinct peaks occurring at the same time in the MEG. These peaks have been extensively verified and observed in EEG experiments. (c) Spatial distribution of the EEG electrodes.

**Figure 7:**
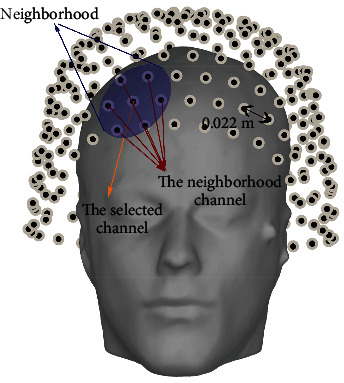
The average distance between sensors and the approximate coverage of the neighbourhood.

**Figure 8:**
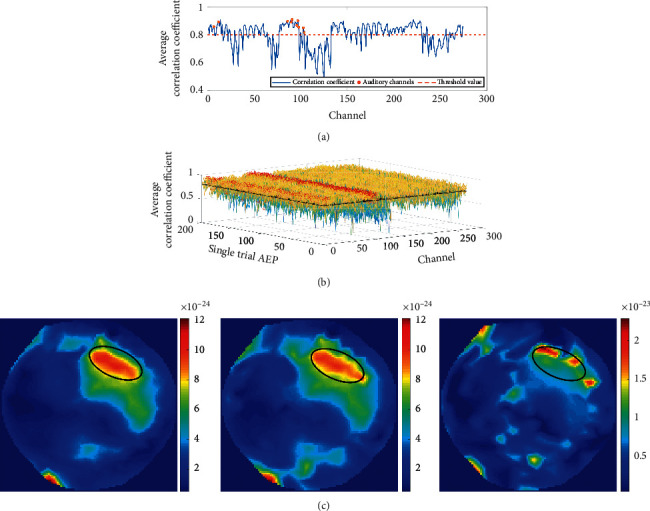
The neighbourhood correlation coefficients calculated from the first dataset. (a) The average neighbourhood correlation coefficient calculated by 200 single trial AEFs. The *x*-coordinate denotes the channel number, and the *y*-coordinate represents the average correlation coefficient between the selected signal and the neighbourhood signals. The red dots indicate 10 sensors located in the auditory part of the temporal lobe, which are all greater than 0.8. (b) All neighbourhood correlation coefficients calculated for 200 single trial AEFs. For each single trial AEF, 274 channels are chosen as the selected channels in turn and the correlation coefficients between them and the neighbourhood signals within their neighbourhood are calculated. The *x*-coordinate represents the channel number, the *y*-coordinate represents single trial, and the *z*-coordinate represents neighbourhood correlation coefficient. The red dots indicate sensors located in the auditory part of the temporal lobe, most of which have neighbourhood correlations greater than 0.8. (c) The effect comparison was performed using different values as threshold. The auditory part of the temporal lobe is demarcated by black circles. The thresholds of correlation coefficients used from left to right are 0.7, 0.8, and 0.9, respectively. The 2D images come from the first single trial AEFs from the first dataset. It can be seen that, for these single trial AEFs, 0.7 and 0.8 have similar effects, but greater than 0.9 is relatively poor.

**Figure 9:**
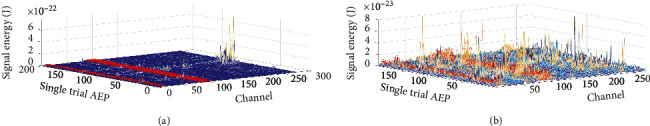
The energy of the single trial AEFs for 274 sensors. (a) The signal energy for each single trail AEF and each channel. For each single trial AEF, the power of 274-channel signals is calculated and displayed. The *x*-coordinate represents the channel number, the *y*-coordinate represents single trial, and the *z*-coordinate represents the signal power. The red dots indicate sensors located in the auditory part of the temporal lobe. In the 93th, 94th, and 112th trials, respectively, MRT31 channel, MRT41 channel, and MRT51 channel detect signals with very high power. (b) The energy of the remaining signals. After deleting the 93th, 94th, and 112th trials' data, the remaining signal energy shows a more random distribution. The red dots indicate signals generated by the auditory part of the temporal lobe.

**Table 1:** Sensors employed in the SQUID-MEG device.

Sensor type	Number	Function
Stimulus channel	1	Recording the electrical trigger signals that produce audio stimuli.
Audio recording channel	1	Recording the audio stimuli sent to the subject.
MEG axial gradiometers	274	Recording the MEG signal.
EEG electrodes	2	Recording the EEG signal.
Electrocardiograph (ECG), bipolar	1	Recording the subject's heartbeat signals.
Electrooculogram (EOG), bipolar	2	Recording the subject's eye movement signals
Head tracking channels	12	Recording the position of the subject's head.

## Data Availability

Three MEG datasets used in this work are obtained from the open database of Brainstorm software, and its website is http://neuroimage.usc.edu/brainstorm.

## Appendix

### A. Average Process for the AEFs

The averaging for the first dataset is exhibited in order to observe some features of the AEFs. The first dataset is 360 s with a sampling rate of 600 Hz and contains 200 single trial AEFs produced by sound stimuli at a frequency of 440 Hz. The average operation for the AEFs mainly includes the following processes.

Data preprocessing: notch filter of 60 Hz, 120 Hz, and 180 Hz, respectively, is performed on the original detected data to remove line frequency noise and its harmonics. Then the positions of heartbeat artifacts and eye movement artifacts are obtained and marked according to heartbeat signals and eye movement signals measured by one bipolar ECG and two bipolar EOG, so that the SSP algorithm can be utilized to remove these artifacts.

Interception for the single trial AEFs: marking single trial AEFs on the MEG according to the occurrence time of stimuli sounds recorded by the audio recording channel. MEG signal segments of 100 ms before and 500 ms after this time point are then intercepted.

Average signal segments: all AEFs are averaged to get the average signal. The AEFs with obvious characteristics can be observed in Figure 6.

### B. Selection of Parameters in the Algorithm

In this work, the neighbourhood radius of 0.022 × 1.7 m and the signal enhancement correlation coefficient threshold of 0.8 are two reasonable parameters obtained through statistics. The average distance between sensors is 0.022 m. The neighbourhood radius selected as 1.7 × 0.022 m ensures that 5 to 8 sensors exist in each selected sensor's neighbourhood, as Figure 7 shows. In other words, 5 to 8 neighbourhood signals are involved in the calculation of correlation coefficient and the competition of signal enhancement.

In addition, the threshold value of correlation coefficient is selected as 0.8. When the correlation coefficient between the neighbourhood signal and the selected signal is greater than 0.8, the neighbourhood signal will be superimposed with the selected signal, so as to enhance the selected signal. The value of 0.8 is selected according to the average correlation coefficient for the selected signal *X*_*i*_^sel^, which can be calculated as

(B.1) $$\begin{array}{c} c_{i}=\frac{1}{m}\sum_{j=1}^{m}c_{ij}. \end{array}$$

For simplicity, we call it the neighbourhood correlation coefficient. *m* represents the number of neighbourhood signals *X*_*j*_^nei^ for the selected signal *X*_*i*_^sel^; *c*_*ij*_ denotes the correlation coefficients between the selected signal *X*_*i*_^sel^ and its neighbourhood signals *X*_*j*_^nei^. 0.8 is obtained based on the statistics of 200 AEFs in the first dataset, as is illustrated in Figure 8. It should be pointed out that because we calculate the correlation coefficients between the neighbourhood signals and average them, they are all positive. But there are also negative correlation coefficients between the MEG signals. There are approximately 10 sensors located in the auditory part of the temporal lobe. For 200 single trial AEFs, 2,000 average correlation coefficients *c*_*i*_ can be obtained, 1711 of which are greater than 0.8 and other values are also close to 0.8. Therefore, the threshold value in the body of the paper is set at 0.8.

### C. Selection of Training Data

In the first dataset, there are 200 single trial AEFs, 3 of which are screened out due to serious pollution caused by noise. This can be observed by the energy of signals of each channel, as is shown in Figure 9(a). In the 93rd, 94th, and 112th trials, there are three channels with very high power, which are MRT31 channel, MRT41 channel, and MRT51 channel, respectively. All these three channels are located closest to the eye, so it can be reasonably speculated that such power fluctuations are due to the incomplete elimination of eye movement artifacts. In the training data, we delete these 3 AEFs. The power of the remaining signals is shown in Figure 9(b).
